# Magnetic NiFe_2_O_4_ Nanoparticles Prepared via Non‐Aqueous Microwave‐Assisted Synthesis for Application in Electrocatalytic Water Oxidation

**DOI:** 10.1002/chem.202101716

**Published:** 2021-08-04

**Authors:** Christopher Simon, Mohamed Barakat Zakaria, Hannah Kurz, David Tetzlaff, André Blösser, Morten Weiss, Jana Timm, Birgit Weber, Ulf‐Peter Apfel, Roland Marschall

**Affiliations:** ^1^ Department of Chemistry University of Bayreuth Universitaetsstrasse 30 95447 Bayreuth Germany; ^2^ Inorganic Chemistry I Ruhr-University Bochum Universitaetsstrasse 150 44801 Bochum Germany; ^3^ Department of Chemistry Faculty of Science Tanta University Tanta 31527 Egypt; ^4^ Fraunhofer Institute for Environmental, Safety, and Energy Technology Osterfelder Strasse 3 46047 Oberhausen Germany

**Keywords:** degree of inversion, microwave synthesis, nanoparticles, oxygen evolution reaction, spinel ferrites

## Abstract

Phase‐pure spinel‐type magnetic nickel ferrite (NiFe_2_O_4_) nanocrystals in the size range of 4 to 11 nm were successfully synthesized by a fast and energy‐saving microwave‐assisted approach. Size and accessible surface areas can be tuned precisely by the reaction parameters. Our results highlight the correlation between size, degree of inversion, and magnetic characteristics of NiFe_2_O_4_ nanoparticles, which enables fine‐tuning of these parameters for a particular application without changing the elemental composition. Moreover, the application potential of the synthesized powders for the electrocatalytic oxygen evolution reaction in alkaline media was demonstrated, showing that a low degree of inversion is beneficial for the overall performance. The most active sample reaches an overpotential of 380 mV for water oxidation at 10 mA cm^−2^ and 38.8 mA cm^−2^ at 1.7 V vs. RHE, combined with a low Tafel slope of 63 mV dec^−1^.

## Graphical abstract

**Phase‐pure spinel‐type** magnetic nickel ferrite (NiFe_2_O_4_) nanocrystals in the size range of 4 to 11 nm were successfully synthesized by a fast and energy saving microwave‐assisted approach. Interestingly, a low degree of inversion is beneficial for the activity in electrocatalytic oxygen evolution.

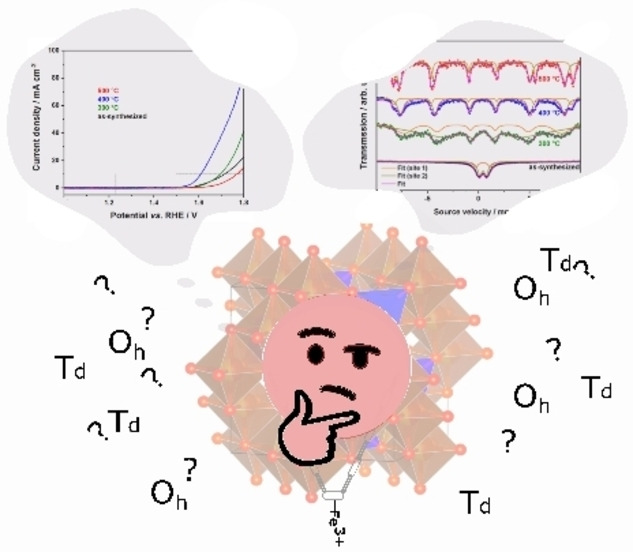

## Introduction

Electrocatalytic water splitting can be regarded as a promising method to store electrical energy in form of hydrogen as a sustainable green energy carrier. However, water splitting typically suffers from the sluggish reaction kinetics of the oxygen evolution half reaction (OER). By employing electrocatalysts, the required overpotential of the OER can be decreased significantly.[^1^, ^2^] However, up‐to‐date benchmark electrocatalysts like RuO_*x*_ or IrO_*x*_ are based on expensive and scarce noble metals, which is the reason why recent research focuses on their substitution.[^3^, ^4^]

Spinel‐type first‐row transition metal oxides (TMOs) or their composite compounds containing nickel, manganese, iron, or cobalt are attractive materials for the electrocatalytic oxygen evolution reaction in alkaline media.[^5^, ^6^, ^7^, ^8^, ^9^] However, Mn‐based electrocatalysts are often neglected due to stability issues.[^10^, ^11^] The use of cobalt is further discussed critically due to its toxicity hazards and limited availability.^[12]^ Therefore, low cost and highly abundant bimetallic NiFe spinel oxides with the elemental composition NiFe_2_O_4_ have gained attention in the field of alkaline water electrolysis, combining remarkable stability in alkaline media with excellent redox properties.[^13^, ^14^] Their ferrimagnetism enables a simple way to recover the electrocatalyst from solution, making the material interesting for future large‐scale industrial applications.^[15]^

NiFe_2_O_4_ is a representative of the spinel group with the general formula AB_2_O_4_. Typically, the inversion parameter *λ* is utilized to describe the particular cationic distributions in tetrahedral and octahedral sites according to the notation [A_1‐*λ*_B_*λ*_]_tet_[A_*λ*_B_2‐*λ*_]_oct_O_4_. According to thermodynamics, the typical arrangement is either a normal spinel with an inversion degree of *λ*=0 (e. g. ZnFe_2_O_4_, CdFe_2_O_4_)[^16^, ^17^] or an inverse spinel with *λ*=1 (e. g. NiFe_2_O_4_, CoFe_2_O_4_),[^18^, ^19^] depending on cationic sizes and crystal field splitting energies. However, deviating situations can be found for non‐equilibrium conditions, for example in the case of nanosized materials.[^20^, ^21^] Thus, values for *λ* of 0.6 and 0.35–0.68 were reported for sol‐gel derived NiFe_2_O_4_ nanomaterials by Atif et al. and Simon et al.,[^22^, ^23^] which is then referred as a partially inverse spinel.

The ferrimagnetic nature of bulk NiFe_2_O_4_ arises from the magnetic moment of anti‐parallel spins between ferromagnetically ordered Fe^3+^ ions (3d^5^, *μ*=5.9) located at tetrahedral voids and ferromagnetically ordered Ni^2+^ (3d^8^, *μ*=2.8) plus Fe^3+^ ions (3d^5^, *μ*=5.9) on octahedral sites. The two sublattices are coupled antiferromagnetically, resulting in a magnetic moment of *μ*=2.8 per formula unit. In case of a normal spinel arrangement of the cations, ferromagnetically ordered Fe^3+^ions (2×3d^5^) would couple antiferromagnetically with the Ni^2+^ (3d^8^) ions resulting in a theoretically expected total spin of *S*=4 (*μ*=8.9) per formula unit, which is significantly higher. Consequently, the magnetic characteristics of NiFe_2_O_4_ strongly depend on the cationic distribution, providing a simple access to tune the magnetic properties of nanosized NiFe_2_O_4_ materials, thus establishing a widespread range of possible magnetic applications, for example in biomedicine^[24]^ or electromagnetic devices.^[25]^

Further, preparation of nanoparticular materials enables high surface‐to‐volume ratios. The increased number of active sites and shortened charge carrier pathways make them attractive for surface‐depending processes like the electrocatalytic water splitting.[^26^, ^27^]

NiFe_2_O_4_ nanoparticles with different morphological characteristics can be prepared by manifold preparation techniques: for example, Zhou et al. prepared NiFe_2_O_4_ nanoparticles with tuneable sizes of 10–120 nm via a low temperature hydrothermal method,^[28]^ whereas Wang et al. chose a solvothermal approach in ethylene glycol producing NiFe_2_O_4_ nanoparticles with adjustable sizes of 7–200 nm.^[29]^ NiFe_2_O_4_ nanoparticles with sizes of 8–28 nm are further available using co‐precipitation methods, which is shown by Maaz et al.^[30]^ Among the various preparation techniques, the applied microwave‐assisted method offers an efficient and controlled heating, shortened reaction times, reproducibility, and improved yields.[^31^, ^32^, ^33^]

Following this experimental procedure, we present a simple one‐step, non‐aqueous microwave‐based synthesis of magnetic NiFe_2_O_4_ nanoparticles, which is also applicable for related compounds such as MgFe_2_O_4_, ZnFe_2_O_4_, and CaFe_2_O_4_.[^34^, ^35^, ^36^, ^37^, ^38^, ^39^] The as‐synthesized powders already contain slightly nanocrystalline NiFe_2_O_4_, while organic residues originating from the metal precursors and the solvent can be removed easily by an additional calcination step at moderate temperatures. Phase‐pure NiFe_2_O_4_ nanoparticles can be prepared by applying the microwave‐based synthesis, with adjustable sizes and surface areas ranging from 4–11 nm and 63–243 m^2^ g^−1^, respectively. The magnetic properties of the obtained materials can be controlled by adjusting the degree of inversion as described above. Thorough characterization of the powders was performed by transmission electron microscopy (TEM), selected area electron diffraction (SAED), X‐ray diffraction (PXRD), Raman spectroscopy, energy dispersive X‐ray spectroscopy (EDXS), nitrogen and water vapor physisorption analysis, diffuse reflectance infrared Fourier transform spectroscopy (DRIFTS), thermogravimetric analysis coupled with online mass spectrometry (TGA‐MS), X‐ray photoelectron spectroscopy (XPS), Mössbauer spectroscopy, and superconducting quantum interference device (SQUID) magnetometry. Additionally, the application potential of the powders was investigated for electrocatalytic oxygen evolution in alkaline media.

## Results and Discussion

Nanocrystalline spinel‐type NiFe_2_O_4_ particles were prepared by a water‐free microwave‐assisted synthesis starting from acetyl acetonate precursors dissolved in *rac*‐1‐phenylethanol as high boiling point solvent. Subsequent thermal treatment was executed to remove organic precursor residues and to adjust the crystallize size precisely. The microwave reaction temperature was set to 225 °C, with subsequent calcination steps at 300, 400, or 500 °C, respectively.

**Figure 1:**
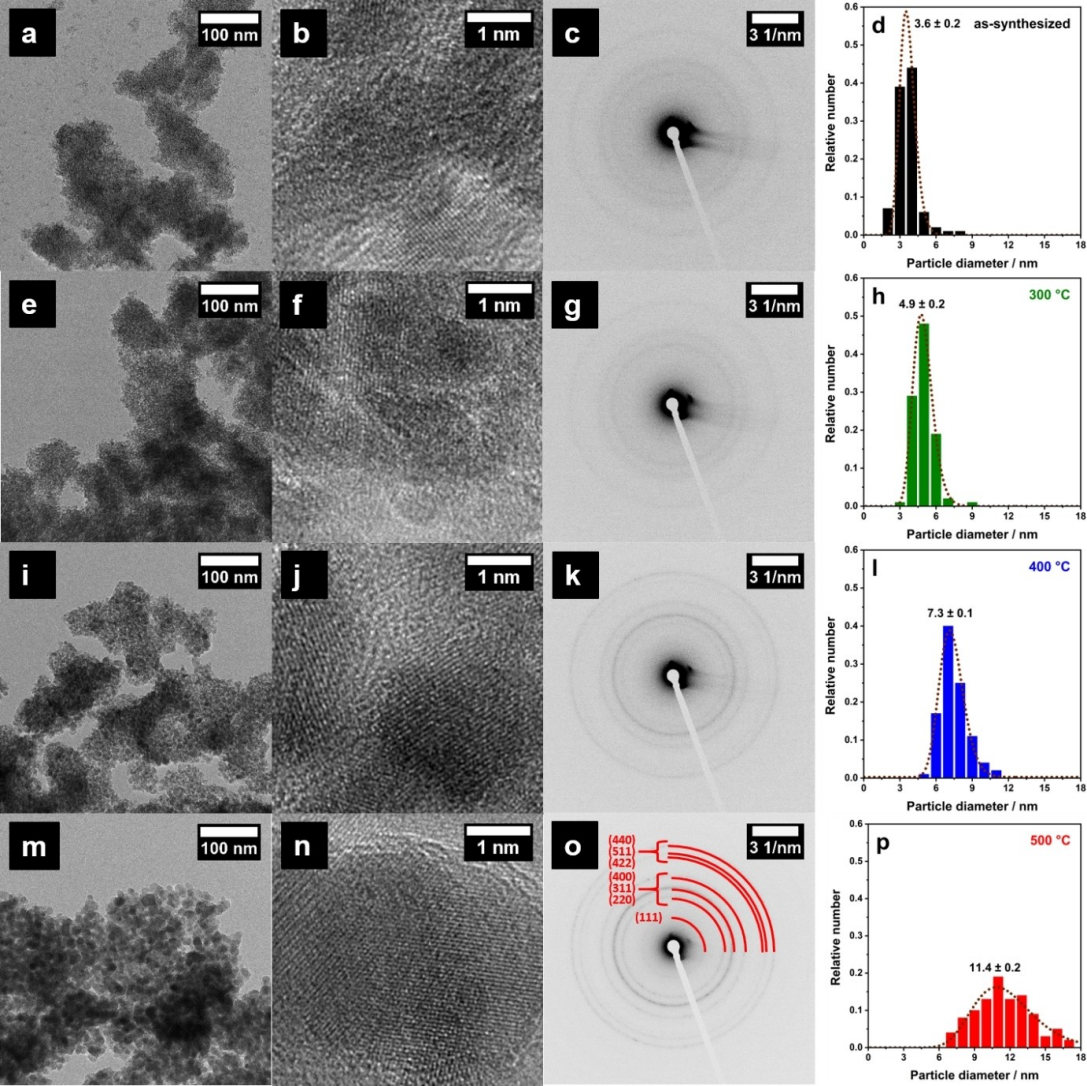
(HR‐)TEM images (first and second column), SAED patterns (third column) plus particle size distributions (right column) of as‐synthesized (a–d) and calcined (300 °C: e–h, 400 °C: i–l, 500 °C: m–p) NiFe_2_O_4_ nanoparticles.

Morphology and crystal structure of as‐synthesized as well as calcined NiFe_2_O_4_ nanoparticles were studied in detail by TEM and SAED (Figure 1). Low‐magnification TEM images generally show randomly shaped particle agglomerates of a few hundred nanometers consisting of small nanoparticles. Hence, the degree of agglomeration is relatively high, which is expected, since no stabilization strategy like surface capping was applied.^[34]^ Lattice planes are visible in the high‐resolution TEM images, even for the as‐synthesized sample. SAED patterns further demonstrate the crystallinity even of the as‐synthesized sample, which is further increasing with the higher calcination temperatures. Patterns of obtained nanoparticles show rings consisting of small dots, each arising from a set lattice planes of an individual nanocrystallite. Upon calcination, the rings/dots are clearly more pronounced, due to the significantly higher crystallinity. Exemplary for the sample calcined at 500 °C, rings were assigned to spinel‐type NiFe_2_O_4_ according to the reference pattern (JCPDS, no. 00‐044‐1485).

Particle size distributions estimated via particle counting can be fitted using a LogNormal function, demonstrating a sharp size distribution for as‐synthesized particles, peaking at 3.6 nm. As expected, particle size distributions get broader when performing a subsequent thermal annealing procedure due to crystallite growth. However, particle size distributions of samples calcined at 300 and 400 °C are still relatively sharp, compared to the 500 °C sample. The averaged nanoparticle size could be estimated to be 4.9 nm (300 °C), 7.3 nm (400 °C), and 11.4 nm (500 °C).

**Figure 2:**
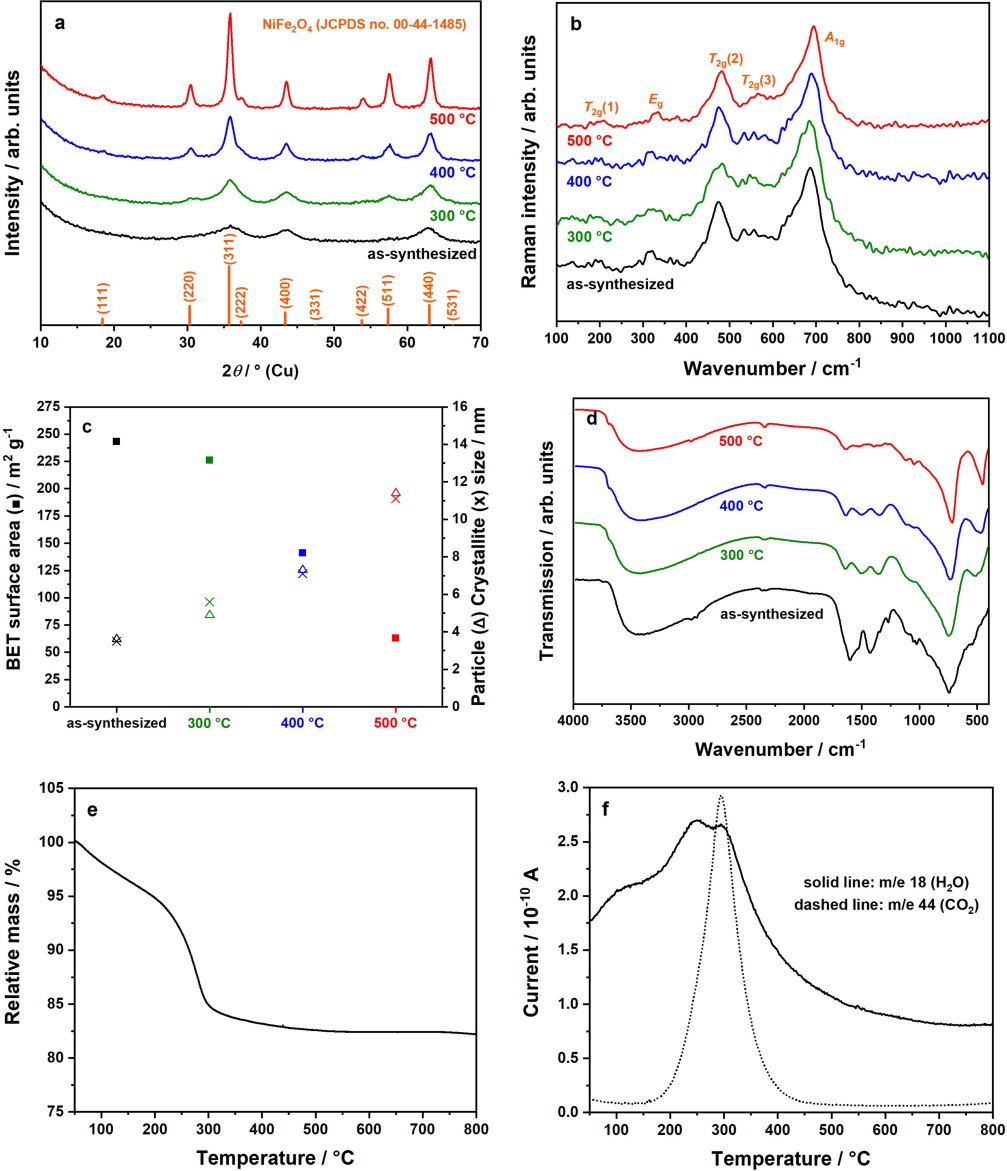
(a) PXRD patterns of calcined samples starting from the as‐synthesized powder prepared at 225 °C via the microwave‐based synthesis strategy, plus (b) corresponding Raman spectra; (c) relations between BET surface area (▪), particle size (Δ), and crystallite size (x); (d) DRIFT spectra of corresponding samples; (e) TG analysis of as‐synthesized sample prepared at 225 °C, plus (f) H_2_O (solid line) and CO_2_ (dashed line) evolution curves during heating monitored by MS.

Syntheses of NiFe_2_O_4_ are sometimes accompanied by the formation of thermodynamically stable *α*‐Fe_2_O_3_ as secondary phase,^[40]^ which can have a significant influence on the material properties. Therefore, a thorough control of phase‐purity by PXRD (Figure 2a), Raman spectroscopy (Figure 2b), and EDXS (Table S1) is indispensable.

Even as‐synthesized samples exhibit relatively wide reflections in the corresponding PXRD pattern that can unequivocally be assigned to spinel‐type NiFe_2_O_4_ with *Fd* $\bar{3}$ *m* space group (JCPDS, no. 00‐044‐1485). By calcination, reflections get sharper, which is consistent with an increased crystallite size. From the integral breadth of the (220), (400), (511), and (440) reflections, the mean crystallite domain sizes could be calculated to 4 nm (as‐synthesized), 6 nm (300 °C), 7 nm (400 °C), and 11 nm (500 °C), demonstrating nanocrystallinity of the samples. These values coincide with the particle sizes obtained from TEM analysis and display the single‐crystalline nature of the NiFe_2_O_4_ nanoparticles.

The phase‐purity of the prepared nanocrystals was additionally investigated by Raman spectroscopy, since small amounts of *α*‐Fe_2_O_3_ might not be detected via PXRD due to the broad reflections caused by the nanosized crystals or the potentially amorphous nature of this oxide. Notably, the cationic distributions in tetrahedral and octahedral sites can also be estimated using this technique if high quality data can be obtained.^[41]^ Figure 2b shows the expected five active Raman modes of spinel‐type NiFe_2_O_4_, even before calcination: *T*_2g_ (200 cm^−1^, 475 cm^−1^, 565 cm^−1^), *E*_g_ (320 cm^−1^), and *A*_1g_ (685 cm^−1^).^[42]^ The most intense *A*_1g_ signal can be assigned to metal‐oxygen symmetric stretching modes located in tetrahedral sites. The additional signals (*T*_2g_, *E*_g_) are due to the symmetric and asymmetric stretching and bending modes of metal‐oxygen units located in octahedral voids.^[43]^ The absence of characteristic Raman modes of hexagonal *α*‐Fe_2_O_3_ also suggests the single‐phase nature of the prepared NiFe_2_O_4_ nanoparticles.^[44]^

The Ni to Fe ratios that were calculated from EDX spectra of the NiFe_2_O_4_ nanoparticles (Table S1, Supporting Information) are close to the expected value of 0.5 for NiFe_2_O_4_, further underlining the single‐phase nature of the prepared nanoparticles. The correct stoichiometry reveals that use of an excess of one precursor component in the synthesis is not necessary, as known for MgFe_2_O_4_.^[36]^ As‐synthesized samples exhibit higher carbon contents compared to the calcined samples, due to remaining organic residues from precursors and solvent.

BET surface areas of NiFe_2_O_4_ nanoparticles are summarized in Figure 2c. The maximum surface area achieved is 243 m^2^ g^−1^ for the as‐synthesized sample, which is an unexpectedly high value for this type of material. As expected, BET surface areas decrease upon calcination because of crystallite growth, which has been proven due to enlarged nanocrystallite sizes. Samples treated at 500 °C still exhibit a high surface area of 63 m^2^ g^−1^.

Further investigations by DRIFTS (Figure 2d) show metal‐oxygen vibrational modes of the spinel ferrite between 540–470 cm^−1^ and 750–740 cm^−1^, respectively. Apparently, these modes are more pronounced after the additional calcination step. For the as‐synthesized samples, two prominent signals are present at 1440–1410 cm^−1^ and 1620–1585 cm^−1^, which can be assigned to carbonyl groups of precursor residues and adsorbed water molecules. Upon calcination at 300–500 °C, these signals mostly disappear, which corresponds to the decomposition of these precursor‐derived surface carbonyl groups. Signals at 2350–2340 cm^−1^, 3500–3000 cm^−1^, and 3700–3690 cm^−1^ can be attributed to carbon dioxide, water, and O−H surface vibrations, respectively. Peaks appearing at 2980–2930 cm^−1^ are due to C−H vibrations.[^36^, ^41^, ^45^]

The presence of surface hydrocarbons is additionally indicated by TGA‐MS of as‐synthesized samples (Figure 2e and f), revealing a significant mass loss of nearly 15 % in the heating range of 250–300 °C. Still, in the range of 300–500 °C samples lose another 2 % of their masses. The first mentioned mass loss originates from the continuing decomposition of remaining organics or carbonates, since H_2_O and CO_2_ traces are detected by in situ MS analysis in exactly this temperature range (Figure 2f).

Conclusively, phase‐pure NiFe_2_O_4_ with tuneable morphology can be prepared by a non‐aqueous microwave‐assisted synthesis, coupled with subsequent thermal annealing step. Aggregated nanocrystals exhibit sizes of 3.6–11.4 nm, resulting in high surfaces up to 243 m^2^ g^−1^.

Compositions of microwave‐derived NiFe_2_O_4_ nanoparticles prepared at 225 °C were investigated via XPS, considering C 1s, O 1s, Ni 2p_3/2_, Fe 2p_3/2_ signals (**Figure S1**). High‐resolution XP spectra of Ni 2p_3/2_ and Fe 2p_3/2_ are complex due to multiplet splitting and shake‐up satellite peaks. The obtained spectra have been fitted with the parameters for NiFe_2_O_4_ as proposed by Biesinger et al.^[46]^ Herein, FWHM and binding energies have been allowed to relax slightly (±0.2 eV for FWHM and ±0.2 eV and 0.4 eV for Fe and Ni binding energies, respectively) in order to compensate for different instrumental resolutions, different pass energies and errors resulting from charge corrections. The measured Ni 2p_3/2_ and Fe 2p_3/2_ spectra are in good agreement with the fits, indicating the presence of NiFe_2_O_4_. O 1s spectra can be fitted with three different peaks at 529.9 eV, 531.4 eV and 533.4 eV, which can be attributed to lattice oxygen, oxygen atoms adjacent to defects, and oxygen from water molecules.^[47]^ The peak at 531.4 eV is decreasing in intensity with increasing annealing temperature, indicating that the amount of defects is reduced by annealing in air.

Elemental compositions obtained from XPS analyses are summarized in Table S2. Compared to the calcined samples, the as‐synthesized powders contain a significantly higher carbon content, because of remaining organic residues. Ni to Fe ratios in the nanocrystals are close to 0.5, which correlates well with previous findings from EDXS.

The described microwave‐based preparation route was also performed at 200, 240, 250, and 275 °C instead of 225 °C, showing that the synthesis procedure works in a temperature range of 200–240 °C (Figure S2). At 250 and 275 °C, the syntheses mostly result in the formation of additional Ni nuclei. Since metallic Ni is an excellent microwave absorbing material, superheating can occur, which is coupled with an unusual pressure increase (Figure S3).^[48]^ Additionally, the time‐dependency of the presented synthesis strategy was studied exemplary for a reaction temperature of 240 °C (the highest possible reaction temperature without Ni nuclei formation and superheating effects). Therefore, the reaction time was varied between 5–30 min. Corresponding PXRD patterns of the as‐synthesized nanopowders are presented in Figure S4. Already after 5 min, extremely wide reflections with low intensities of spinel‐type NiFe_2_O_4_ are observable, demonstrating very small crystallites already forming after this short reaction time. With increasing reaction times, samples become more and more crystalline, which is demonstrated by the increasing crystallite sizes and decreasing BET surface areas (Figure S5). Comparable microwave‐based experiments were performed for ZnFe_2_O_4_ spinels by Dolcet et al.,^[37]^ obtaining highly crystalline phase‐pure ZnFe_2_O_4_ nanoparticles already after 5 min of reaction time. Due to restricted yields at 200 °C and the potential danger of superheating at 240 °C, the standard reaction temperature was chosen to be 225 °C for full analysis.

**Figure 3:**
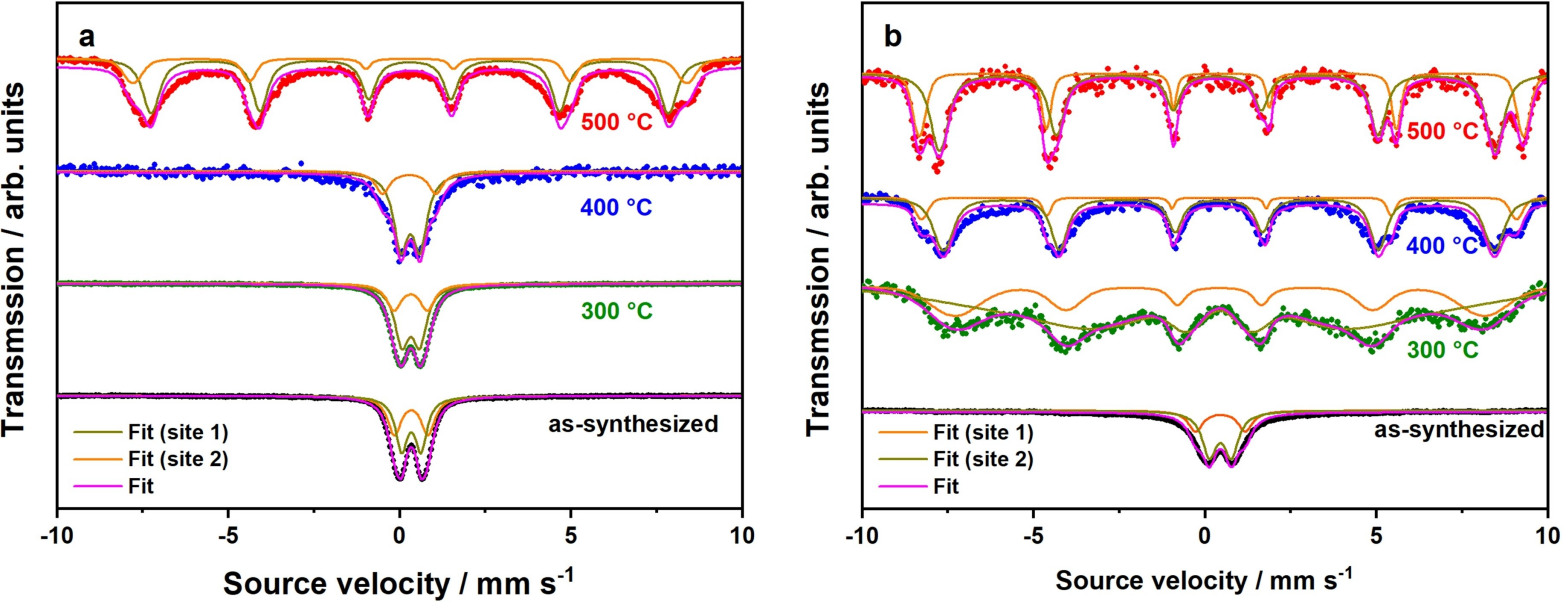
^57^Fe Mössbauer spectra of NiFe_2_O_4_ nanoparticles taken at 298 K (a) and 80 K (b). Generally, data points were fitted for both Fe^3+^ cations located in tetrahedral (site 1) and octahedral (site 2) voids, giving the overall fit.

In the thermodynamically most stable state, the crystal structure of NiFe_2_O_4_ can be described as inverse spinel with an inversion degree of *λ*=1. The anti‐parallel spin arrangement of cations located in tetrahedral and octahedral sites results in the overall ferrimagnetic nature of the Neél collinear type.[^49^, ^50^] Non‐equilibrium conditions can be induced by preparing nanosized NiFe_2_O_4_ materials, resulting in a deviation of cationic distributions (0<*λ*<1), which is then referred as partially inverse spinel. Cationic distributions of such iron‐based spinels can be estimated by ^57^Fe Mössbauer spectroscopy, which was performed at 298 K and 80 K for microwave‐derived NiFe_2_O_4_ nanoparticles prepared at 225 °C (Figure 3). For the inverse spinel NiFe_2_O_4_ with a permanent magnetic moment, a magnetic hyperfine splitting into two sextets is expected, corresponding to Fe^3+^ cations located in tetrahedral and octahedral voids, respectively. At room temperature, Mössbauer spectra of the nanoparticles depend strongly on the synthesis conditions. The as‐synthesized sample shows two very similar doublets with an isomer shift *δ* of 0.35 mm/s (both) and a quadrupole splitting Δ*E*_Q_ of 0.98 and 0.55 mm/s, respectively,^[51]^ indicating the presence of Fe^3+^ in tetrahedral and octahedral sites and superparamagnetic behavior. A reliable assignment of the doublets to the different sites and thus determination of λ is not possible with the data available. A subsequent thermal annealing step at temperatures of 300 or 400 °C does not change the described situation significantly. A calcination temperature of 500 °C is necessary to observe a sextet due to the magnetic hyperfine splitting in the room temperature Mössbauer spectrum. The increased particle size results in a reduced fluctuation of the magnetization and the magnetization reversal is now slow compared to the time resolution of the Mössbauer spectrum. Please note that the blocking temperature for the magnetization reversal determined by magnetic measurements, which will be shown below, is lower due to the different time scales of the two methods. Notably, the sextet is a superposition of two individual sextets arising from Fe^3+^ cations located in tetrahedral and octahedral sites. The above findings suggest changing cationic distributions and blocking temperatures by varying nanoparticle sizes. From the relative areas of these sextets, the inversion degree *λ* can be determined approximately, by equalizing the areas (tetrahedral, octahedral) with the term *λ*/(2‐*λ*).^[52]^ For this purpose, measurements were repeated at 80 K. Here, the sextets become observable for the three calcined samples, showing that the blocking temperatures are above 80 K on the Mössbauer time scale. The estimation of *λ* is only meaningful for samples calcined at 400 and 500 °C. For the sample calcined at 300 °C the small particle size leads to a significant broadening of the lines due to the iron centers at the surface that prevent a reliable determination of the areas. Obtained values for *λ* (400 °C : 0.30, 500 °C : 0.66) suggest the presence of partially inverse spinels and further underline the strong relation between nanoparticle size and cationic distributions. A higher calcination temperature results in a more inverse NiFe_2_O_4_ spinel, which is in agreement with thermodynamics. Thereof, cationic distributions of NiFe_2_O_4_ nanoparticles are easily adjustable by the chosen calcination temperature. Further details obtained from ^57^Fe Mössbauer spectroscopy (isomer shifts, quadrupole splittings, internal magnetic fields) are summarized in Table S3.

**Figure 4:**
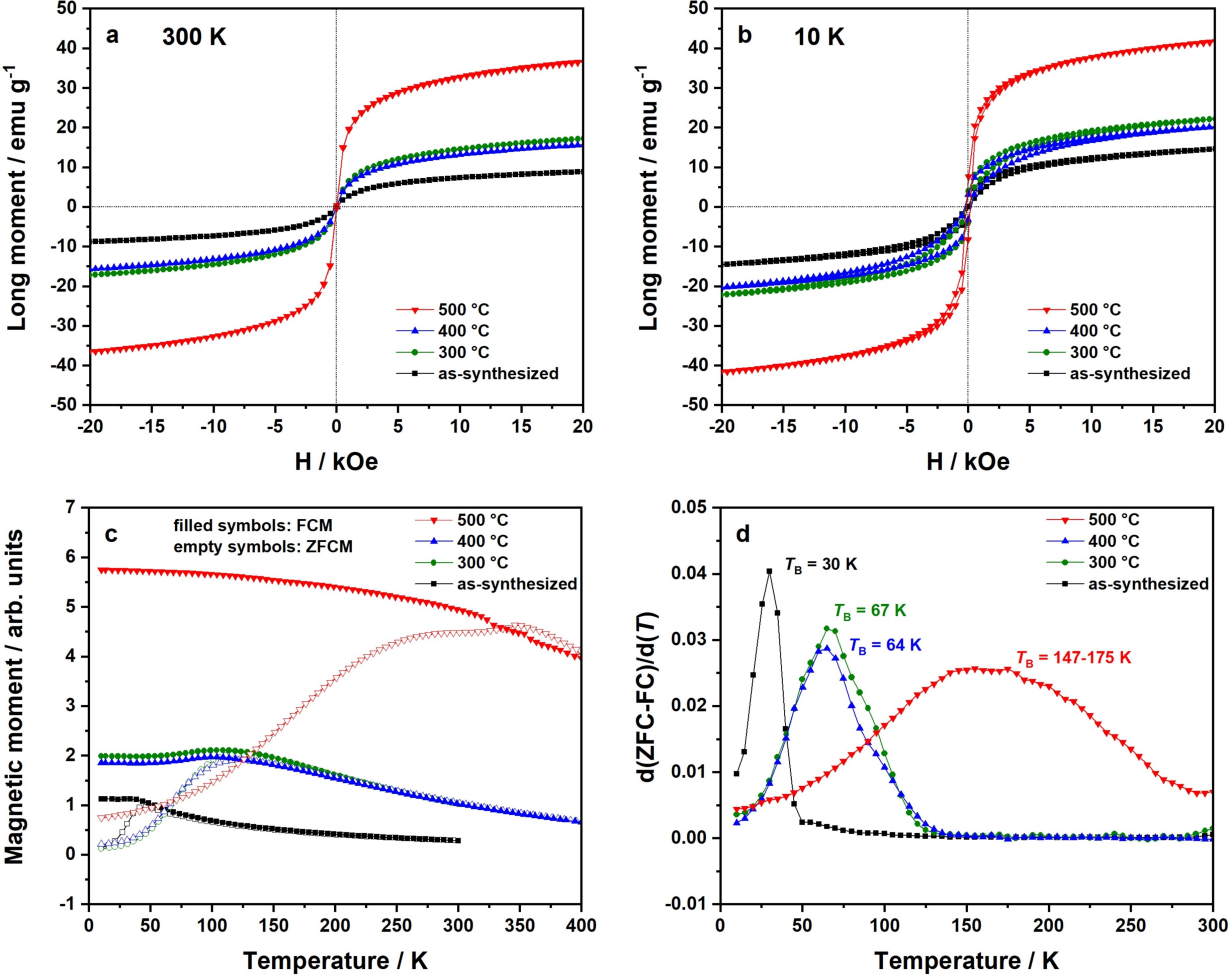
Mass loss corrected magnetization curves of NiFe_2_O_4_ nanoparticles at 300 K (a) and 10 K (b), measured via SQUID magnetometry. The field‐cooled (FCM) (applied field 100 Oe) and zero‐field‐cooled magnetization (ZFCM) curves are shown in (c), the corresponding d(ZFC‐FC)/d(T) plots in (d). Here, the local maxima represent the blocking temperatures.

Magnetization curves of NiFe_2_O_4_ nanoparticles measured at 300 K and 10 K obtained by SQUID magnetometry are presented in Figure 4a and b. To include the mass portion of diamagnetic components, original data sets were corrected by 22.0 % (as‐synthesized), 3.6 % (300 °C), 1.5 % (400 °C), and 0.8 % (500 °C), which is in accordance with TGA analysis (cf. Figure 2e). Uncorrected magnetization plots are additionally presented in Figure S6. The saturation magnetizations *M*_S_ (with/without mass correction) are further summarized comparatively in Table 1. Determined *M*_S_ values are generally higher for the 10 K measurements compared to the room temperature measurements, indicating that complete saturation is not reached at room temperature. Additionally, magnetization curves taken at 10 K feature a narrow hysteresis, in line with the determined blocking temperature that is for all samples above 10 K. Comparing the differently treated samples, the saturation magnetizations estimated at 300 K and 10 K increase with the calcination temperature by tendency. The as‐synthesized particles exhibit a saturation magnetization of 14.6 emu g^−1^, compared to 41.8 emu g^−1^ of samples treated at 500 °C. This is in line with the increase in particle size. Effects like surface spins, spin canting, spin glass structures, and dead layers, depending mainly on the size of NiFe_2_O_4_ nanoparticles, may contribute to the significantly changed, for smaller particles usually reduced, magnetic properties.^[53]^ However, for samples treated at 300 and 400 °C a deviation from this trend is observed; the saturation magnetization after calcination at 300 °C is higher (22.2 emu g^−1^) than after calcination at 400 °C (20.3 emu g^−1^) and does not go in line with the particle size. This indicates a change in the inversion degree already at those temperatures, thus *λ* (300 °C)<*λ* (400 °C).

**Table 1:** Saturation magnetization at 20 kOe obtained from SQUID magnetometry experiments. The assumed unit cell is Ni_8_Fe_16_O_32_ with M=1875.05 g mol^−1^.

	size/nm	300 K			10 K		
		*M*_s_/emu g^−1^	*M*_s_ (corr)/emu g^−1^	*M*_s_/Kemu mol^−1^	*M*_s_/emu g^−1^	*M*_s_ (corr)/emu g^−1^	*M*_s_/Kemu mol^−1^
as‐synthesized	3.6	7.2	8.8	16.6	11.9	14.6	27.3
300 °C	4.9	16.6	17.2	32.3	21.4	22.2	41.6
400 °C	7.3	15.5	15.7	29.5	20.0	20.3	38.0
500 °C	11.4	36.3	36.6	68.5	41.4	41.8	78.3

Using Neel's sublattice theory, the calculated saturation magnetization of bulk NiFe_2_O_4_ (thermodynamically stable state) is 50 emu g^−1^.^[54]^ According to *M*_S_=*N*_A_ *μ*_B_ *S*, this value strongly depends on the expected total spin per formula unit, a reduced *λ* will lead to an increased saturation magnetization. With *λ* =0.66 after calcination at 500 °C and a particle size of 11.4 nm, a comparison with the achieved saturation magnetization indicates that near‐bulk magnetization is reached despite the small nanocrystals size of NiFe_2_O_4_. A relation of the magnetic properties with both, inversion degree and nanoparticle size, is therefore obvious. Thus the magnetism of ultrasmall NiFe_2_O_4_ nanoparticles is reduced due to the large particle surface, but the changed cationic distributions can counteract this effect, which was shown already for NiFe_2_O_4_[^55^, ^56^] and other spinel‐type materials, like MgFe_2_O_4_ ^[36]^ or CoFe_2_O_4_.^[57]^

The field‐cooled (FCM) and zero‐field‐cooled (ZFCM) magnetization curves are depicted in Figure 4c. Blocking temperatures *T*_B_ could be estimated via the d(ZFC‐FC)/d(*T*) plots shown in Figure 4d. ZFCM‐FCM plots of magnetic NiFe_2_O_4_ as‐synthesized nanoparticles and those calcined at 300 °C and 400 °C show the expected behavior for soft magnetic nanomaterials with narrow particles size distributions.^[58]^ However, ZFCM‐FCM curves exhibit differences depending on the particle size. The d(ZFC‐FC)/d(*T*) plot of the as‐synthesized powder is characterized by a sharp signal with a maximum at 30 K, giving the mandatory temperature for the spin flip, which is referred as blocking temperature *T*_B_. Thermal treatment results in a signal broadening, which correlates to the loss of particles size homogeneity (cf. Figure 1d, h, l, p) and an increasing degree of magnetic anisotropy. Blocking temperatures of calcined samples could be estimated to 67 K (300 °C), 64 K (400 °C), and 147–175 K (500 °C). For the particles calcined at 500 °C the signal is very broad indicating a significantly broader particle size distribution. A relation between blocking temperature and calcination temperature/particle size is obvious. When performing Mössbauer spectroscopy at 80 K (cf. Figure 3b), the blocking temperature is near or above 80 K for all calcined samples, resulting in sextets due to magnetic hyperfine splitting. Since as‐synthesized particles feature a blocking temperature of 30 K, only a doublet due to superparamagnetism can be observed at 80 K.

To conclude, particle size, crystallite size, surface area, and following cationic distributions can easily be controlled by the synthesis conditions. Consequently, the magnetic properties of nanoparticular NiFe_2_O_4_ strongly depend on the subsequent thermal annealing procedure. By adjusting particle sizes precisely, inversion degree and magnetic characteristics of obtained NiFe_2_O_4_ nanomaterials can be tailored for any particular application.

Alongside the magnetic applications, NiFe_2_O_4_ is a promising material to catalyze the oxygen evolution reaction, combining remarkable surface areas (in case of nanoparticle or mesoporous morphology) with an outstanding stability in alkaline media. For electrochemical oxygen evolution to take place at NiFe_2_O_4_ nanoparticles, the presence of a preferably bulk hydrophilicity and wettability is beneficial.^[59]^ Therefore, water vapor physisorption isotherms of NiFe_2_O_4_ nanoparticles prepared at 225 °C were recorded (Figure S7a) and for better comparison corrected with regard to the respective specific BET surface area (Figure S7b). For a metal oxide, NiFe_2_O_4_ nanocrystal powders generally exhibit relatively high water vapor uptakes of 0.77 cm^3^ m^−2^ (as‐synthesized), 1.00 cm^3^ m^−2^ (300 °C), 1.63 cm^3^ m^−2^ (400 °C), and 2.16 cm^3^ m^−2^ (500 °C) at a relative pressure of 0.95 (95 % humidity).^[60]^ Thus, a higher calcination temperature results in a higher overall water uptake, which can be correlated to the decomposition of hydrophobic carbon surface residues (cf. Figures 2e and f). From this point of view, the calcination of NiFe_2_O_4_ powder further promotes the applicability in electrochemical water splitting.

**Figure 5:**
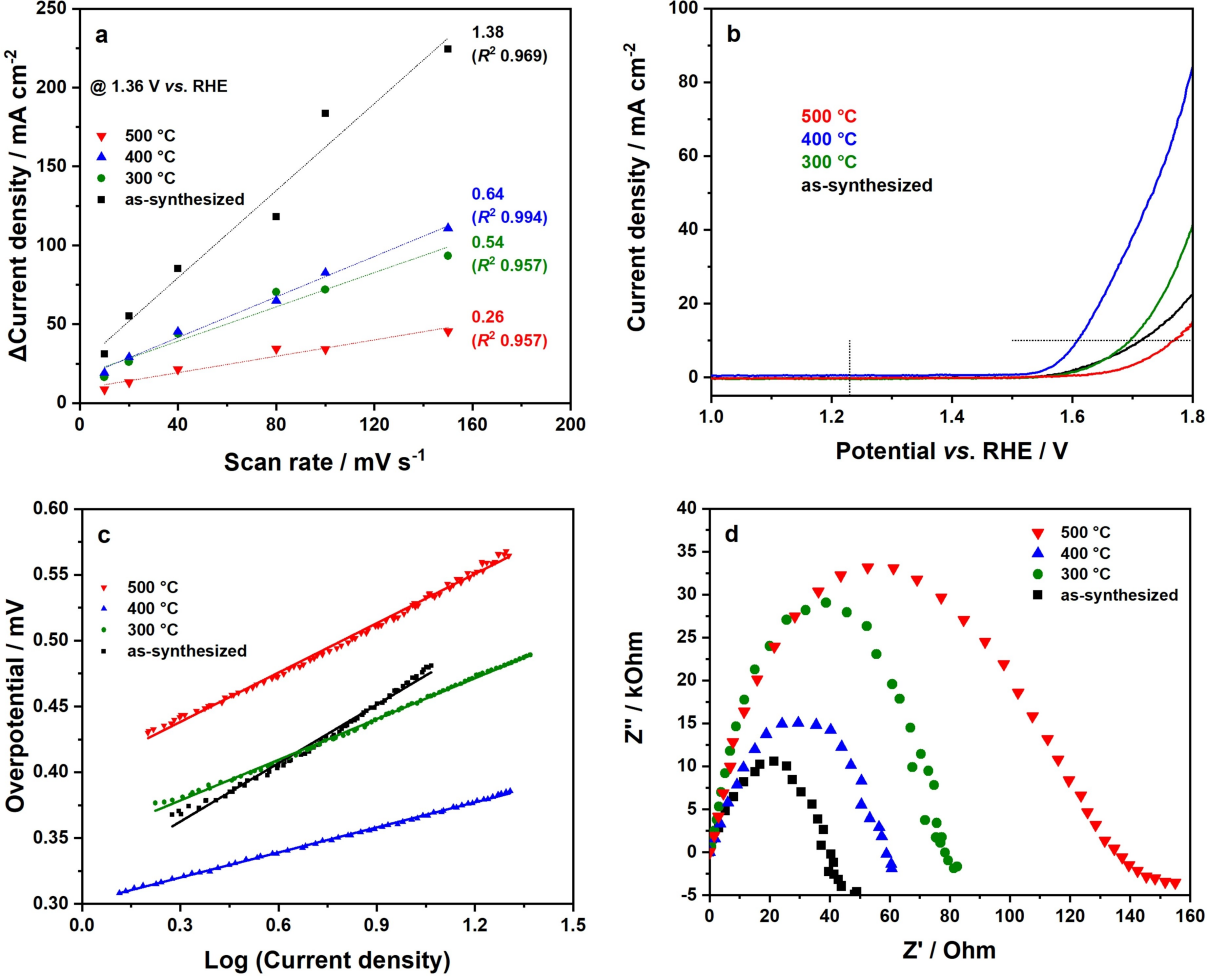
Electrochemical characterization of microwave‐derived NiFe_2_O_4_ nanoparticles prepared at 225 °C. (a) Estimation of the electrochemical double‐layer capacitance c_DL_ as slope of the linear fit, which is proportional to ECSA, (b) Linear sweep voltammograms (LSV) without iR compensation measured with a scan rate of 50 mV s^−1^ in 1 M KOH, corresponding Tafel plots are further shown in (c). EIS Nyquist plots of electrodes at applied potential (1.7 V vs. RHE) are presented in (d).

The ferrimagnetism of spinel‐type NiFe_2_O_4_ enables a simple method to recover the electrocatalyst for catalyst reuse. Due to nanostructuring, an improved electrical conductivity is ensured, which is also beneficial for electrocatalysis.[^61^, ^62^] Hence, the application potential of NiFe_2_O_4_ nanocrystals for the oxygen evolution reaction (OER) was investigated, examining the influence of surface area and crystallinity on the overall activity in detail. For this purpose, a glassy carbon (GC) electrode modified with 0.35 mg cm^−2^ NiFe_2_O_4_ was used as working electrode. Experiments were performed under alkaline conditions (1 M KOH). To compare surface areas obtained by physisorption measurements with the corresponding electrochemical active surface area (ECSA), cyclic voltammetry data measured at various scan rates in the non‐faradaic region (1.31–1.41 V vs. RHE) were considered (Figure S8). Differences of forward and backward scans at 1.36 V vs. RHE were plotted against the scan rate (mV s^−1^). Linear extrapolation of the data points gives the electrochemical double‐layer capacitance *c*_DL_ as slope. Normalization on the surface area of the GC electrode (0.071 cm^2^) and division of *c*_CL_ with the specific capacitance (*c*_S_, typical value 0.03 mF cm^2^ for alkaline solutions) finally gives the electrochemical active surface area (Figure 5a).[^3^, ^63^, ^64^] Parameters obtained from electrochemical measurements are further summarized in Table 2. As‐synthesized nanoparticles exhibit an electrochemical active surface of 3.27 cm^2^. Upon calcination, ECSA values decrease, which can be correlated to the enlarged crystallite size. These observations go in line with a decreased BET surface area (cf. Figure 2c). Comparing as‐synthesized and 500 °C samples, the electrochemical active surface decreases by 81 % (3.27 cm^2^ vs. 0.62 cm^2^), comparable to the observed BET surface area change by likewise 73 % (243 m^2^ g^−1^ vs. 63 m^2^ g^−1^). Interestingly, samples treated at 300 and 400 °C exhibit comparable ECSA. Linear sweep voltammograms (LSVs) of NiFe_2_O_4_‐modified glassy carbon working electrodes presented in Figure 5b reveal a dependency of the OER activity and morphology. The current densities read at 1.7 V vs. RHE are 8.7 mA cm^−2^ (as‐synthesized), 10.8 mA cm^−2^ (300 °C), 38.8 mA cm^−2^ (400 °C), and 3.4 mA cm^−2^ (500 °C). Accordingly, NiFe_2_O_4_ nanoparticles calcined at 400 °C show the highest OER activity. Apart from that, the as‐synthesized and 300 °C samples perform worse compared to the sample calcined at 400 °C. Thus, observed activities partly mismatch the estimated electrochemical double‐layer capacitances, providing that other factors also influence the activity for the electrocatalytic water oxidation. The reason for the relatively low OER activity of the uncalcined sample is the organic surface coating, which originates from precursor (acetylacetonate) and solvent (*rac*‐1‐phenylethanol) residues, decreasing the surface hydrophilicity significantly. Notably, a calcination temperature of 300 °C is not sufficient to remove these organic surface layers, contrary to 400°, which is reflected by the significantly increased OER performance at 400 °C. Interestingly, the breakdown in activity towards the sample calcined at 500 °C not only correlates with a lower BET surface area, but also with the doubling of the degree of inversion observed from Mössbauer spectroscopy (0.30 to 0.66, cf. Figure 3). For NiFe_2_O_4_ nanoparticles, a more inverse spinel seems to lower the activity for the electrocatalytic oxygen evolution reaction in alkaline media. Such an influence of *λ* on the OER activity was earlier observed for spinel manganese ferrite (MnFe_2_O_4_) nanoparticles.^[65]^ Since both magnetic properties and OER activity are therefore correlated to the inversion degree, *λ* can be considered as one key factor to establish a function‐tailored spinel‐type material.

**Table 2:** Parameters obtained from electrochemical investigations on NiFe_2_O_4_ nanoparticles prepared at 225 °C. The overpotential for the oxygen evolution reaction was read at a current density of 10 mA cm^−2^.

	Overpotential/mV	Tafel slope/mV dec^−1^	ECSA/cm^2^
as‐synthesized	487	148	3.27
300 °C	464	103	1.28
400 °C	380	63	1.52
500 °C	539	125	0.62

To sum up, a thermal annealing step at moderate temperatures like 400 °C results in the maximum activity, but higher calcination temperatures result in a breakdown of activity, which is influenced by a lower surface area^[66]^ and an increased degree on inversion. Overpotentials (at 10 mA cm^−2^) of 487 mV (as‐synthesized), 464 mV (300 °C), 380 mV (400 °C), and 539 mV (500 °C) are acceptable values for the electrocatalytic water oxidation, and further go in line with previous results. From this point of view, an energy‐consuming annealing step at temperatures higher than 400 °C is not recommended for a high electrocatalytic activity. The potential‐dependent kinetics of the oxygen evolution reaction (OER) with NiFe_2_O_4_ nanoparticles were studied via Tafel analysis (Figure 5c). Tafel slopes of 148 mV dec^−1^ (as‐synthesized), 103 mV dec^−1^ (300 °C), 63 mV dec^−1^ (400 °C), and 125 mV dec^−1^ (500 °C) reveal favorable kinetics for the oxygen evolution reaction, especially for nanoparticles calcined at 400 °C. In addition, electrochemical impedance spectroscopy on material electrodes was performed (Figure 5d). NiFe_2_O_4_ nanoparticles calcined at 500 °C possess a relatively high resistance, which could be correlated to the slightly increased crystallite size but can ultimately be correlated to the larger degree of inversion of the spinel as well as to their altered magnetic behavior, resulting in a decreased activity for electrocatalytic water oxidation. When the calcination temperature is reduced to 400 °C, the resistance is much lower, which can be correlated to the remarkably better performance of the corresponding sample. The sample calcined at 300 °C shows a similar resistance comparable to samples calcined at 400 and 500 °C, although the particle size is smaller. However, a carbon layer on the nanoparticle surface seems to influence their electrocatalytic behavior. The as‐synthesized particles exhibit the lowest resistance of all samples, possibly due to the smallest particle surface, which contradicts to the worse OER performance. However, the four samples show quite small Nyquist arcs (50–140 Ω), indicating a fast charge transfer at the interface of electrode and electrolyte for high performance.

For future industrial applications, stable performances over longer periods of time are essential, combined with high activities. Therefore, a chronoamperometric long‐term stability test was performed with the most active 400 °C‐treated sample. Therefore, a constant potential of 1.56 V vs. RHE was applied, which is near to the experimental onset (overpotential) of the sample (Figure S9). In the first three hours, it is obvious that there is a significant increase in the current density, which might be attributed to the electrochemical activation of the electrode, which were not conditionalized by cyclic voltammetry prior to chronoamperometry. After the activation process, a fast current density drop can be observed, before reaching a relatively constant level after around 10 hours. After one day, the current density is reduced to 38 %, compared to the start. A part of the current density loss originates possibly from the detachment of the catalyst from the electrode surface during the long‐term stability test. Notably, after the course of the experiment, fine particles could be observed in the electrolyte visually.

To outline the electrochemical measurements, microwave‐derived NiFe_2_O_4_ nanoparticles synthesized at 225 °C are able to electrocatalyze the oxygen evolution reaction. The overall performances are strongly influenced by the subsequent thermal annealing procedure, with samples treated at 400 °C being the most active nanocrystals, reaching an overpotential of 380 mV for water oxidation. Thus, a high surface area combined with a low crystallinity and suitable bulk hydrophilicity seem to be key factors for a high performance in the oxygen evolution reaction. However, a temperature treatment at 400 °C is necessary to achieve the maximum activity. In fact, temperature treatment below 400 °C or omitting the calcination step entirely results in smaller particle sizes, but carbon residues originating from the synthesis procedure are not decomposed completely, hindering the electrocatalytic water oxidation.

## Conclusions

We introduced a non‐aqueous microwave‐assisted sol‐gel synthesis for the preparation of NiFe_2_O_4_ nanocrystals. The reaction operates in a temperature window of 200–240 °C, yielding crystalline nanopowders directly after the synthesis. By a subsequent thermal annealing step at 300–500 °C, the morphology of the nanoparticles can be tuned preciselyand their surface area is also cleaned from residual hydrocarbon impurities. Applying for example a reaction temperature of 225 °C, phase‐pure spinel‐type NiFe_2_O_4_ nanoparticles with sizes of 3.6–11.4 nm and surface areas 63–243 m^2^ g^−1^ can be produced.

Due to the preparation of such ultrasmall NiFe_2_O_4_ nanocrystals, non‐equilibrium site occupations can be found, which is indicated by changed cationic distributions. The structure changes from a complete inverse spinel (thermodynamically stable state) to a partially inverse spinel upon nanostructuring. Due to the changed occupation of tetrahedral and octahedral sites in the NiFe_2_O_4_ nanoparticles, the magnetic properties can be tailored precisely by adjusting the degree of inversion. Thus, NiFe_2_O_4_ nanocrystals are applicable for a widespread range of magnetic applications.

In addition, NiFe_2_O_4_ nanoparticles were tested for their ability to perform the electrocatalytic oxygen evolution reaction in alkaline media. Keeping the composition of the materials constant, the influence of morphology, crystallinity, and degree of inversion on the activity was investigated, revealing that heating above 400 °C is not necessary to get high electrochemical activity. Moreover, we can correlate the breakdown in activity at higher calcination temperatures to the higher degree of inversion of the spinel crystal. The most active sample, which was treated at 400 °C, reaches a remarkable activity (overpotential 380 mV, Tafel slope 63 mV dec^−1^). It is worth to mention that in context of recent literature about NiFe_2_O_4_ in water oxidation electrocatalysis, and although we used unmodified and phase‐pure NiFe_2_O_4_ nanoparticles, our as‐synthesized materials exhibit a comparable performance (Table S4). No strategies like faceting,^[67]^ modification with other compounds,^[68]^ surface functionalization,^[69]^ or introduction of vacancies^[70]^ were applied, underlining the high potential of our facile synthesis to prepare active electrocatalysts and showing the importance of the inversion degree, *λ* on the overall performance.

## Experimental section

For each synthesis of NiFe_2_O_4_ nanoparticles, 128.5 mg (0.5 mmol) Ni(acac)_2_ (*SigmaAldrich*, for synthesis) and 353.2 mg (1 mmol) Fe(acac)_3_ (*Acros Organics*, +99 %) were dissolved in 15 mL of *rac*‐1‐phenylethanol (*SigmaAldrich*, 98 %) using ultrasonication. The obtained solution was transferred into a 30 mL borosilicate microwave glass vessel. The solution was then heated as fast as possible to a maximum temperature of 200–275 °C (standard 225 °C) for typically 30 min using microwave irradiation (Anton Paar Monowave 400 equipped with MAS24 autosampler) under constant stirring (600 rpm). Next, the reaction mixture was cooled down to 55 °C using compressed air. To study the influence of the reaction time, the reaction was performed additionally for 5–25 min (5 min steps) at 240 °C. Obtained nanoparticles were precipitated with *n*‐pentane and washed for three times with water/acetone mixtures (3 : 1, 12 : 1, 12 : 1) and diethyl ether. The resulting powder was dried at 80 °C overnight. To remove remaining organic residues and to adjust the crystallite size, the as‐synthesized samples were further calcined in a Nabertherm muffle furnace under air atmosphere at 300/400/500 °C (heating rate 5 °C/min, holding time 5 h).[^34^, ^37^]

### Characterization techniques

Transmission electron microscopy (TEM) and selected area electron diffraction (SAED) were performed on a 200 kV JEOL‐JEM‐2200FS EFTEM with Schottky FEG and In‐Column Omega Energyfilter from JEOL GmbH. For sample preparation, samples were dispersed in ethanol (*Merck, LiChrosolv®, gradient grade for liquid chromatography*), before dropping a small amount of the dispersion on a carbon film coated Cu TEM grid (200 Mesh). Images were edited using ImageJ 1.52a.

X‐ray powder diffraction (PXRD) was measured with a Malvern PANalytical Empyrean device equipped with a PixCel 1D detector using Cu Kα radiation (wavelengths *λ*_1_=1.54046 Å and *λ*_2_=1.54439 Å). Acceleration voltage and emission current were set to 40 kV and 40 mA, respectively. Data were collected in a range of 10–70° 2*θ*, with a step size of 0.144° and a scan step time of 883 s. To minimize X‐ray fluorescence, detector pulse height distribution (PHD) settings were changed to 8.05 keV (lower level) and 11.27 keV (upper level). From PXRD data, the crystallite domain size could be estimated using the integral breadth *β*.^[71]^ Therefore, 2*Θ* values were transformed to units of *s* (*s*=(2sin*Θ*/*λ*). Then, dividing the area under an individual reflection by its height gives the integral breadth *β*_hkl_, which is the reciprocal of the crystallite size *L*_hkl_.

Raman spectra were collected on a LABRAM I Raman spectrometer from Horiba Jobin Yvon GmbH, additionally modified with an Olympus BX41 microscope (50x magnification). A HeNe laser (*λ*=632.82 nm) was utilized, operated with a power of 2 mW. For each sample, three spots were measured with 15 s integration time and 10 co‐additions. Subsequently, individual spectra were averaged, normalized and smoothed using a FFT filter. Data were measured within a range of 100–1000 cm^‐1^ with a step size of 1.02 cm^‐1^.

Energy‐dispersive X‐ray spectroscopy (EDXS) experiments were performed using a Zeiss Leo 1530 instrument equipped with an UltraDry‐EDX detector (ThermoFisher Scientific NS7). A working distance of 8 mm was applied, while the acceleration voltage was set to 15 kV in order to detect Ni, Fe, and O. Prior to the analysis, samples were sputtered with 1.3–2 nm of Pt with a Cressington Sputter Coater. For each sample, four points were investigated, before averaging the values, with the standard deviation as error bar.

Diffuse reflectance infrared Fourier transform spectroscopy (DRIFTS) was measured on Bruker alpha II device within a range of 400–4000 cm^−1^, applying a spectral resolution of 10 cm^−1^ and 200 co‐additions for a single scan.

Surface areas were estimated with N_2_ at 77 K using an Anton Paar QUADRASORB evo surface area & pore size analyzer. For surface area evaluation, the BET (Brunauer‐Emmett‐Teller) theory was applied. Prior to the measurements, samples were degassed in vacuum at 120 °C for 12 h to remove surface adsorbed water.

Volumetric H_2_O vapor physisorption measurements were performed with the ASiQMP‐MP‐AG setup (Anton Paar QuantaTec, Boynton Beach, USA) at 20 °C and constant *p*_0_=2317.67 Pa (17.384 Torr). The samples were also degassed at 120 °C for 12 h prior to the measurement.

Thermogravimetric analysis (TGA) in synthetic air was carried out on a Netsch Jupiter STA 449 C thermo‐balance, additionally coupled with a Netzsch Aeolos QMS 403 C mass spectrometer (MS). Data were collected in a range of 30–1000 °C with a heating rate of 5 °C min^−1^. MS traces of H_2_O (*m*/*e* 18) and CO_2_ (*m*/*e* 44) were recorded.

X‐ray photoelectron spectroscopy (XPS) was performed with a Physical Electronics PHI VersaProbe III Scanning XPS Microprobe equipped with a monochromatic aluminum Kα source in the KeyLab “Device Engineering” of the Bavarian Polymer Institute (BPI). Survey scans were measured with a pass energy of 224 eV, a step size of 0.8 eV and a time of 50 ms per step, whereas high‐resolution spectra were recorded with a pass energy of 26 eV, a step size of 0.1 eV and a step time of 50 ms; beam diameter was set to 100 μm for all measurements. All samples were flooded with low energy electrons and argon ions to prevent surface charging. Argon ion sputtering before measuring was omitted to prevent reduction of iron and nickel species.^[47]^ The recorded data was evaluated with CasaXPS using Gaussian‐Lorentzian GL(30) line shapes and Shirley backgrounds; the C 1s signal was set to 284.8 eV for charge correction.

^57^Fe Mössbauer spectroscopy at room temperature and 80 K was performed on a SeeCo constant acceleration spectrometer with temperature controller, equipped with a ^57^Co source in a Rh matrix. Isomer shifts are referred to *α*‐Fe at room temperature. Data sets were fitted with the WMOSS program by applying a Voigt profile‐based (hyperfine field distribution) or Lorentzian profile‐based (quadrupole doublets) least‐square routine.

Magnetic experiments were performed on a superconducting quantum interference device (SQUID) MPMS‐XL5 device from Quantum Design. Samples were measured in gelatin capsules held in a plastic straw. Field measurements at 10 and 300 K were performed from 0.1 to 20 to −20 kOe in the hysteresis mode (step width 0.5 kOe). The scans were corrected for the diamagnetism of the sample holder and the sample mass loss during calcination, which was estimated via TGA. ZFCM/FCM (zero‐field‐cooled/field‐cooled magnetization) curves were collected at 0.1 kOe from 10 to 300/400 K and back to 10 K in the sweep mode with a velocity of 5 K min^−1^. For this purpose, samples were first cooled in the SQUID cavity without applying an external magnetic field.

For OER measurements, a conventional three‐cell with a glassy carbon electrode‐coated with the catalyst was used as working electrode, saturated calomel electrode (SCE) as reference electrode, and platinum wire as counter electrode. For preparing the working electrodes, 5 mg of the catalyst was dispersed in a water/ethanol mixture (950 μL, 3 : 1 v/v) under sonification for 30‐minutes, followed by adding Nafion (50 μL, 5 wt %) with a sonification process for another 1 h to afford a homogeneous ink. The ink was dropped onto the surface on the glassy carbon electrode with a geometric area of 0.071 cm^2^ and dried at room temperature. LSV was performed at a potential range between 0.0 V and 1.0 V vs. SCE for the OER with a scan rate of 50 mV s^−1^ in 1 M KOH. The overpotential was determined at a current density of 10 mA cm^−2^. The electrochemical impedance spectroscopy (EIS) was performed at 0.7 vs. SCE from 0.01 to 20 kHz. The cyclic voltammetry (CV) data were collected at the potential range from 0.3 to 0.4 V vs. SCE at different scan rates (10, 20, 40, 80, 100, and 150 mV s^−1^) to calculate the electrochemical double‐layer capacitance, which is proportional to ECSA. The collected potential *versus* the reversible hydrogen electrode (RHE) was calculated according to the following formula: E(RHE)=E(SCE)+0.241 V+0.059 V*pH.

## Conflict of interest

The authors declare no conflict of interest.

## Supporting information

As a service to our authors and readers, this journal provides supporting information supplied by the authors. Such materials are peer reviewed and may be re‐organized for online delivery, but are not copy‐edited or typeset. Technical support issues arising from supporting information (other than missing files) should be addressed to the authors.

Supporting Information
