# Supplementary material for: Magnetic NiFe2O4 Nanoparticles Prepared via Non‐Aqueous Microwave‐Assisted Synthesis for Application in Electrocatalytic Water Oxidation
Source: Chemistry. 2021 Aug 4;27(68):16990–7001. doi: 10.1002/chem.202101716 (PMC9291896; doi:10.1002/chem.202101716)
Supplement: Supplementary file 1 — Supporting Information [file CHEM-27-16990-s001.pdf]

Chemistry–A European Journal

Supporting Information

Magnetic $NiFe_2O_4$ Nanoparticles Prepared via Non-Aqueous Microwave-Assisted Synthesis for Application in Electrocatalytic Water Oxidation

Christopher Simon, Mohamed Barakat Zakaria, Hannah Kurz, David Tetzlaff, André Blösser, Morten Weiss, Jana Timm, Birgit Weber, Ulf-Peter Apfel, and Roland Marschall*

Table S 1: Elemental compositions of $NiFe_2O_4$ nanoparticles prepared at 225 °C with the microwave-assisted synthesis strategy, estimated *via* EDXS. From each sample, four spots were investigated, showing averaged values with standard deviations as error bars.

	C / at %	O / at %	Fe / at %	Ni / at %	Ni:Fe ratio
as-synthesized	13.5 (5.9)	54.2 (6.5)	22.5 (5.3)	9.6 (1.6)	0.43 (0.03)
300 °C	9.5 (2.2)	51.0 (1.6)	27.7 (1.9)	11.8 (0.8)	0.42 (0.00)
400 °C	5.5 (0.9)	52.1 (2.1)	29.3 (1.9)	13.2 (0.7)	0.45 (0.03)
500 °C	7.7 (3.7)	46.8 (2.4)	31.8 (4.5)	13.8 (1.5)	0.44 (0.02)

Table S 2: Elemental compositions of $NiFe_2O_4$ nanoparticles prepared at 225 °C with the microwave-assisted synthesis strategy, estimated *via* XPS.

	C / at %	O / at %	Fe / at %	Ni / at %	Ni:Fe ratio
as-synthesized	29.0	48.3	15.1	7.5	0.50
300 °C	23.4	50.1	17.8	8.7	0.49
400 °C	23.5	50.5	17.7	8.3	0.47
500 °C	23.1	49.4	18.6	9.0	0.48

Table S 3: Parameters obtained from ^{57}Fe Mössbauer spectroscopy.

		298 K				80 K			
	fit	δ / mm s^{-1}	ΔE_Q / mm s^{-1}	B_0 / T	*Rel. Area*	δ / mm s^{-1}	ΔE_Q / mm s^{-1}	B_0 / T	*Rel. Area*
as-synthesized	1	0.35	0.98	-	0.45	0.46	1.47	-	0.32
	2	0.35	0.55	-	0.55	0.46	0.63	-	0.68
	overall	0.35	0.64	-		0.46	0.66	-	
300 °C	1	0.33	0.98	-	0.32	0.43	-	48.2	0.26
	2	0.33	0.49	-	0.68	0.43	-	-	0.74
	overall	0.33	0.55	-		0.43	-	47.6	
400 °C	1	0.32	1.56	-	0.24	0.46	-	54.1	0.15
	2	0.32	0.53	-	0.76	0.46	-	50.0	0.85
	overall	0.32	0.54	-		0.46	-	50.8	
500 °C	1	0.28	-	50.4	0.25	0.36	-	54.8	0.33
	2	0.28	-	47.0	0.75	0.36	-	50.2	0.67
	overall	0.28	-	47.5		0.36	-	54.1	

Table S 4: Comparison of the oxygen evolution performance of microwave-derived $NiFe_2O_4$ nanoparticles from this work with other recently reported spinel $NiFe_2O_4$ electrocalalysts. Overpotentials are estimated at a current density of 10 mA cm^{-2}.

description	synthesis	calcination	overpotential	Tafel slope	electrolyte	ref.
particles	hydrothermal	800 °C	570 mV	93 mV/dec	0.1M KOH	[1]
fibres	solution blow spinning	800 °C	433 mV	134 mV/dec	1.0M KOH	[2]
mesoporous	soft templating	400 °C	410 mV	50 mV/dec	1.0M KOH	[3]
nanoparticles (O deficient)	hydrothermal, H_2 treatment	-	389 mV	64 mV/dec	1.0M KOH	[4]
particles	sol-gel	700 °C	381 mV	47 mV/dec	1.0M KOH	[5]
nanorods	thermal decomposing	350 °C	342 mV	44 mV/dec	1.0M KOH	[6]
nanoparticles	sol-gel	500 °C	290 mV	42 mV/dec	1.0M KOH	[7]
quantum dots	electro-deposition	-	262 mV	37 mV/dec	1.0M KOH	[8]
this work	microwave	400 °C	380 mV	63 mV/dec	1.0M KOH	-

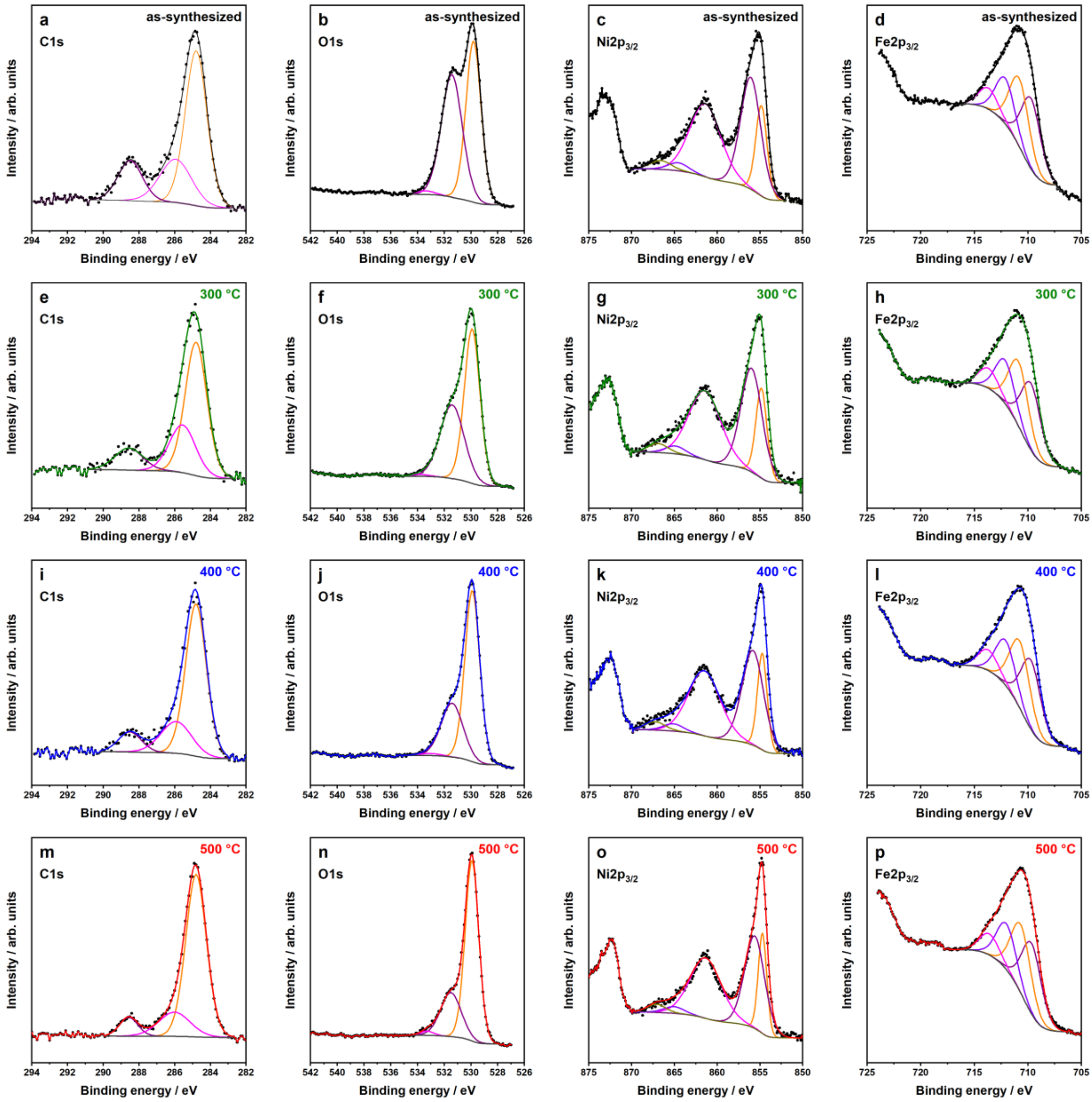

Figure S1: XP spectra fits of the C 1s, O 1s, Ni 2p$_{3/2}$, plus Fe 2p$_{3/2}$ core levels of as-synthesized (a-d) and calcined (300 °C: e-h, 400 °C: i-l, 500 °C: m-p) $NiFe_2O_4$ nanoparticles.

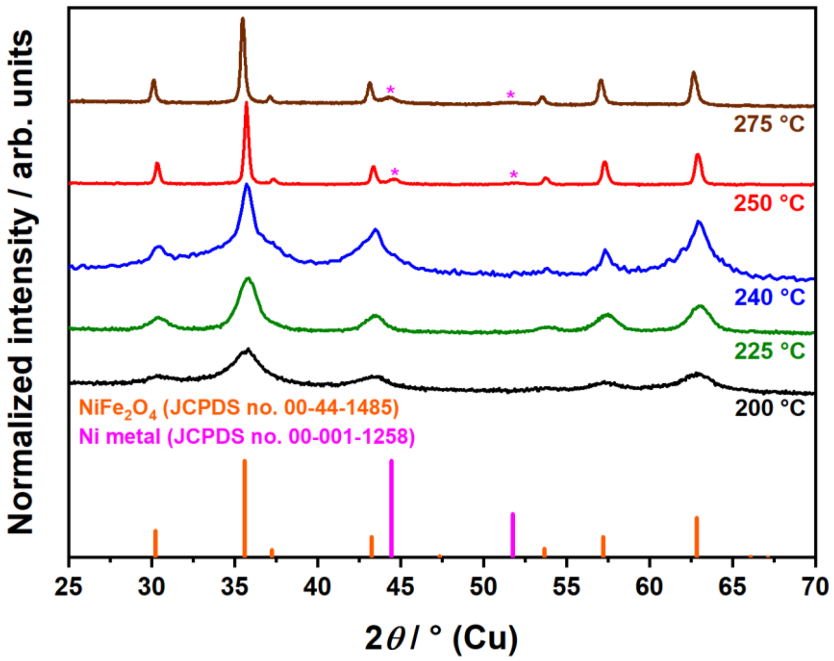

Figure S2: PXRD patterns of as-synthesized $NiFe_2O_4$ nanoparticles prepared at different temperatures *via* the microwave-based synthesis. For better comprehensibility, intensities were normalized with respect to the most intensive reflection at 35.6 ° 2*θ.* At 250 and 275 °C, a Ni metal by-phase can be observed. Here, Ni nuclei form during microwave treatment. Since metallic Ni is an excellent microwave absorbing material, superheating occurs, coupled with an unusual pressure increase. Another result of superheating is an increased crystallinity, which is reflected by sharpened reflections. The possible temperature range of the synthesis is therefore 200-240 °C.

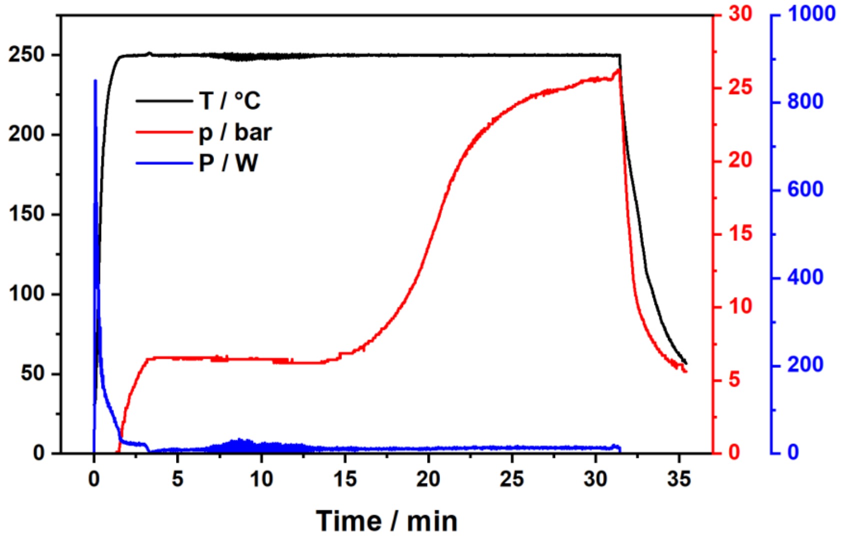

Figure S3: Microwave data (temperature measured *via* IR sensor, evolved pressure, and applied power) of a run performed at 250 °C. The unusual pressure increase beginning after approx. 15 min origins in superheating processes due to metallic Ni deposits, which are excellent absorbers of microwave irradiation. At the standard reaction temperature of 225 °C, the pressure stays constant during the whole reaction time.

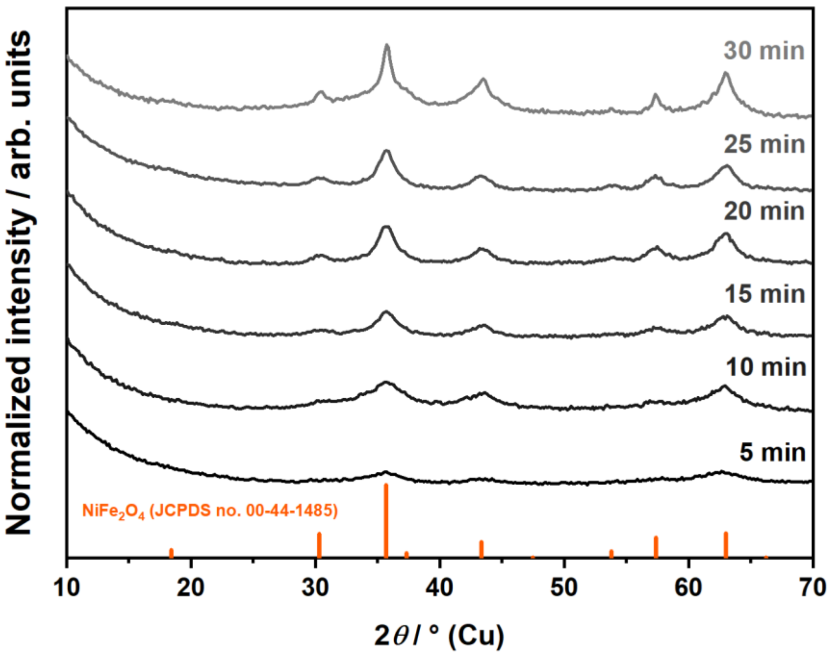

Figure S4: PXRD pattern of as-synthesized $NiFe_2O_4$ nanoparticles prepared at a temperature of 240 °C in the microwave-based synthesis. For better comprehensibility, intensities were normalized with respect to the most intensive reflection at 35.6 ° 2*θ*. In this experiment, the reaction time was changed in a range of 5-30 min within steps of 5 min.

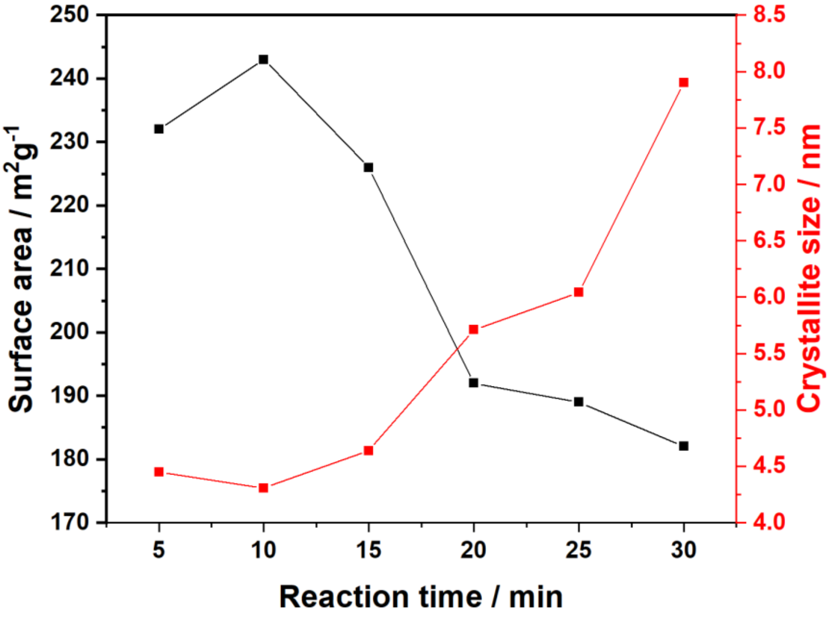

Figure S5: Surface areas determined *via* BET analysis of nitrogen physisorption data and crystallite sizes of as-synthesized $NiFe_2O_4$ nanoparticles prepared at 240 °C. The reaction time was changed in a range of 5-30 min within steps of 5 min.

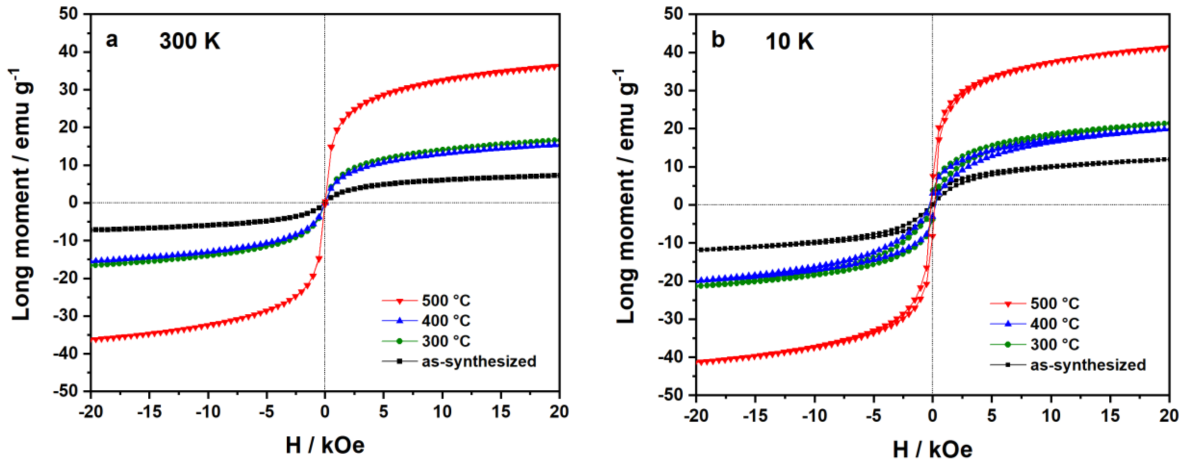

Figure S6: Uncorrected magnetization curves of $NiFe_2O_4$ nanoparticles at 300 K (a) and 10 K (b), measured *via* SQUID magnetometry.

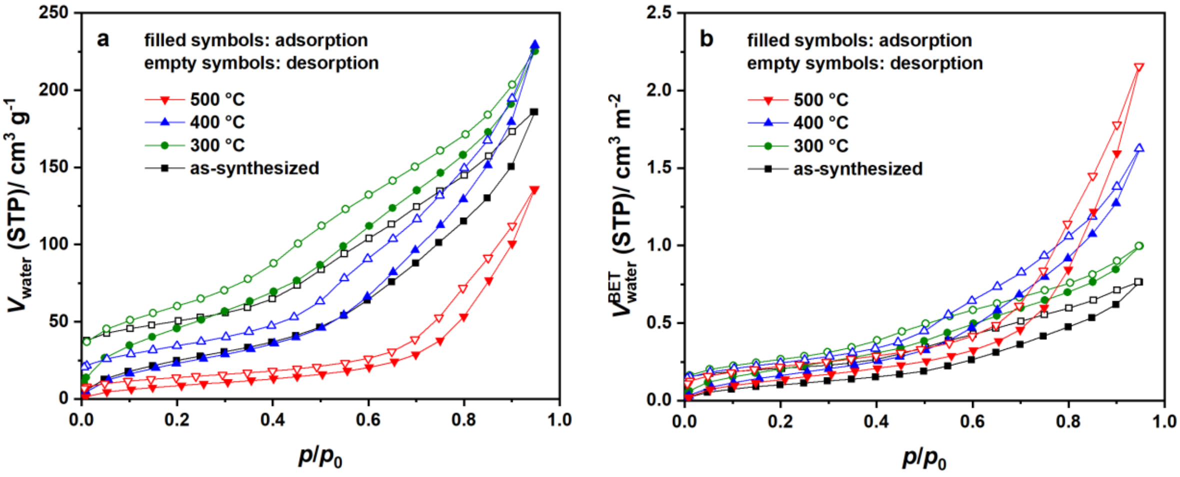

Figure S7: (a) Water vapor physisorption isotherms of $NiFe_2O_4$ nanoparticles prepared at 225 °C and (b) respective surface area-normalized isotherms. Water vapor physisorption experiments were performed at 20 °C.

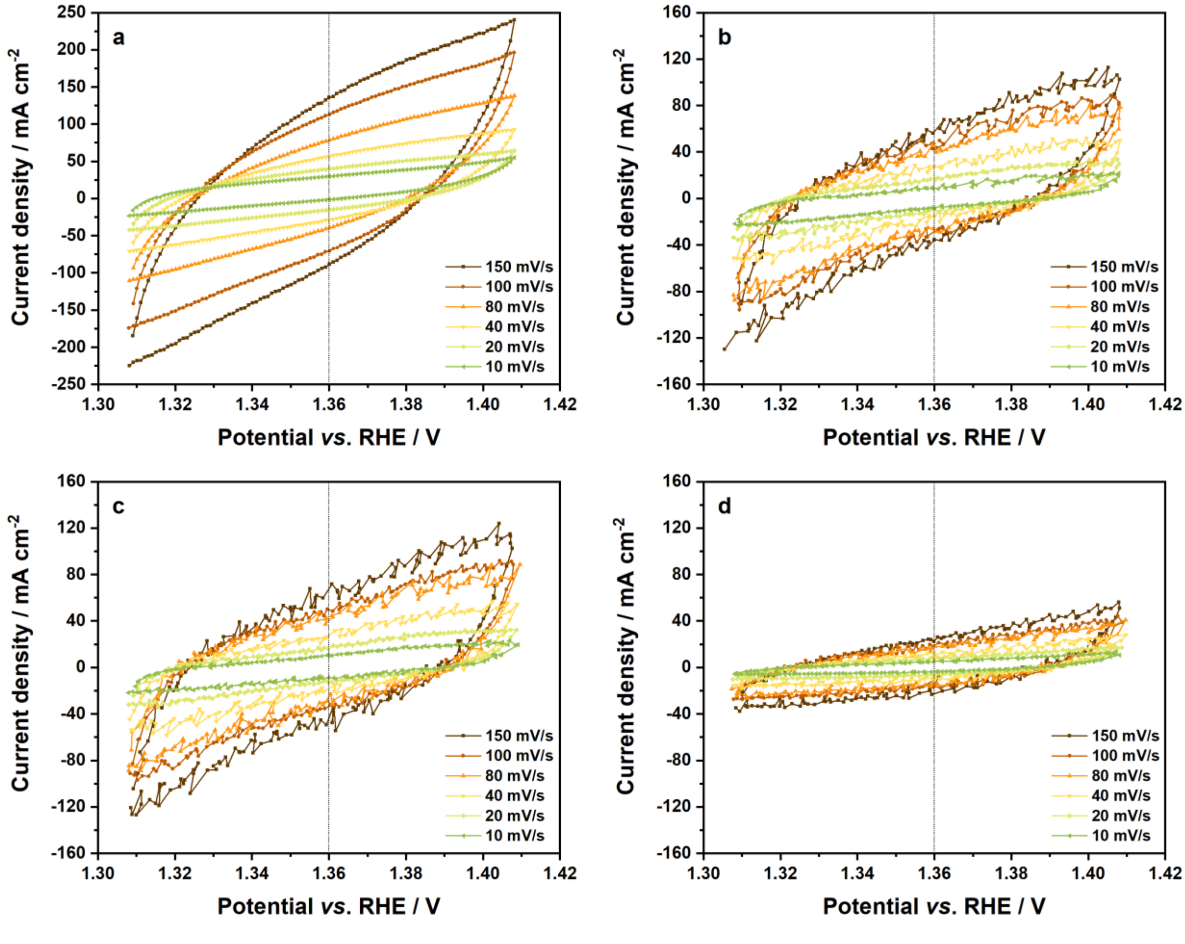

Figure S8: Static cyclic voltammetry curves (CVs) measured at scan rates of 150, 100, 80, 40, 20, and 10 mV s^{-1} for as-synthesized (a) plus 300 °C (b), 400 °C (c) and 500 °C (d) calcined $NiFe_2O_4$ nanoparticles. From the data sets, the electrochemical double-layer capacitance c_{DL} could be estimated, which is proportional to the electrochemical active surface area (ECSA).

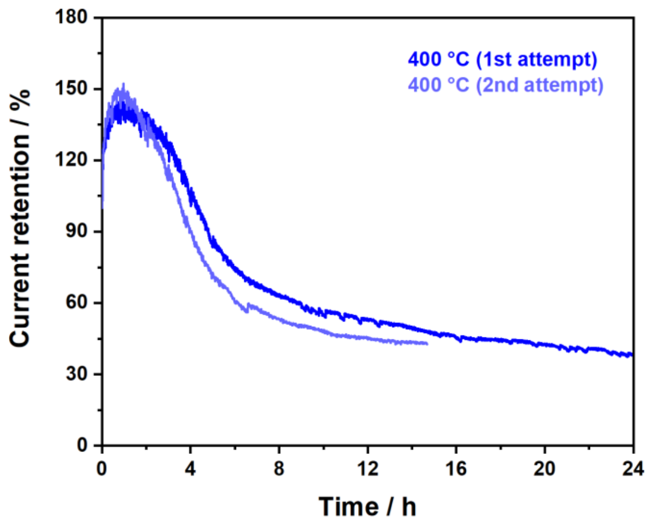

Figure S9: Chronoamperometric long-term stability tests at applied potential (1.56 V *vs*. RHE) with two different electrodes in 1M KOH solution. For both experiments, the most active sample (calcination at 400 °C) was considered.

Additional literature

[1] S. Pal, U. P. Azad, A. K. Singh, D. Kumar, R. Prakash, *Electrochim. Acta* **2019**, *320*, 134584.

[2] V. D. Silva, L. S. Ferreira, T. A. Simões, E. S. Medeiros, D. A. Macedo, *J. Colloid Interface Sci.* **2019**, *540*, 59–65.

[3] C. Simon, J. Timm, D. Tetzlaff, J. Jungmann, U.-P. Apfel, R. Marschall, *ChemElectroChem* **2021**, *8*, 227–239.

[4] D. Lim, H. Kong, N. Kim, C. Lim, W. Ahn, S. Baeck, *ChemNanoMat* **2019**, *5*, 1296–1302.

[5] V. Maruthapandian, M. Mathankumar, V. Saraswathy, B. Subramanian, S. Muralidharan, *ACS Appl. Mater. Interfaces* **2017**, *9*, 13132–13141.

[6] G. Liu, K. Wang, X. Gao, D. He, J. Li, *Electrochim. Acta* **2016**, *211*, 871–878.

[7] N. Dalai, B. Mohanty, A. Mitra, B. Jena, *ChemistrySelect* **2019**, *4*, 7791–7796.

[8] H. Yang, Y. Liu, S. Luo, Z. Zhao, X. Wang, Y. Luo, Z. Wang, J. Jin, J. Ma, *ACS Catal.* **2017**, *7*, 5557–5567.
